# Metabolic Heterogeneity Confers Differences in the Tumor Microenvironment of Aggressive Types of Melanomas

**DOI:** 10.1111/jop.70042

**Published:** 2025-08-27

**Authors:** Juliana de Souza do Nascimento, João Figueira Scarini, Erika Said Abu Egal, Marcelo Brum Corrêa, Rodrigo Ribas Dias dos Reis, Luciana Schultz Amorim, Rachel Martins Marinho Robim, Clóvis Antônio Lopes Pinto, Patricia Maria Peresi, Ana Lucia Noronha Francisco, Felipe Paiva Fonseca, Luiz Paulo Kowalski, Román Carlos, Fernanda Viviane Mariano, Albina Altemani

**Affiliations:** ^1^ Department of Pathology, Faculty of Medical Sciences University of Campinas (UNICAMP) São Paulo Brazil; ^2^ Department of Oral Diagnosis School of Dentistry, University of Campinas (FOP/UNICAMP) São Paulo Brazil; ^3^ Biorepository and Molecular Pathology University of Utah (UU) Salt Lake City Utah USA; ^4^ Head and Neck Surgery Department, Oncology Center (CEON), Fornecedores de Cana Hospital, Piracicaba São Paulo Brazil; ^5^ Institute of Pathological Anatomy São Paulo Brazil; ^6^ Department of Pathology, A. C. Camargo Cancer Center São Paulo Brazil; ^7^ Departament of Pathology, School of Dentistry University of Minas Gerais (UFMG) Belo Horizonte Brazil; ^8^ Department of Head and Neck Surgery and Otorhinolaryngology, A. C. Camargo Cancer Center São Paulo Brazil; ^9^ Pathology Section, Centro Clínico de Cabeza y Cuello/Hospital Herrera Llerandi Guatemala City Guatemala

**Keywords:** cutaneous melanoma, glycogenesis, lipogenesis, metabolism, oral melanoma, sinonasal melanoma

## Abstract

**Background:**

Melanoma affects skin and mucosa and can be particularly aggressive when the lesion is an advanced cutaneous tumor or located in the sinonasal or oral mucosa. Reprogramming of energy metabolism has been defined as a hallmark of cancer; so this study aimed to verify the expression of proteins related to metabolism and cellular proliferation.

**Methods:**

Immunohistochemical analysis with antibodies adipophilin, FASN, GLUT‐1, HIF‐1α, and Ki‐67 was performed in a series of 28 sinonasal melanomas (SM), 16 oral melanomas (OM), and 39 cutaneous melanomas (CM). For CM, 25 cases with matched lymph node metastases were analyzed, while 17 mucosal and 15 cutaneous melanocytic nevi served as controls.

**Results:**

SM showed an increased frequency of undifferentiated cells, necrotic areas, and marked expression of adipophilin in comparison to OM. In metastatic CM, a significant increase of FASN expression was detected. However, the frequency of expression of this protein was not significantly different between primary tumors and their metastasis. Concerning adipophilin expression in CM with or without metastasis, no significant difference was found, whereas the Ki‐67 proliferative index was significantly lower in metastatic tumors. Benign melanocytic lesions showed lower expression of all markers.

**Conclusion:**

SM and OM show marked differences in metabolic phenotype alterations since SM are more frequently positive for adipophilin. In CM, the marked expression of FASN in metastatic tumors suggests that these proteins probably contribute to disease progression.

## 1 Introduction

Melanoma, a malignant neoplasm of melanocytic origin, affects skin and mucosa and can be particularly aggressive when the lesion is an advanced cutaneous tumor [1] or located in the sinonasal [2] or oral mucosa [3]. The estimates of the American Cancer Society for melanoma in the United States for the year 2025 are about 104 960 new cases of melanoma and 8430 deaths [4]. Aggressive morphologic features, such as undifferentiated small round cell predominance, extensive necrosis, vascular, and deep tissue invasion are more frequently seen in sinonasal melanomas (SM) than in oral melanomas (OM) and cutaneous melanoma (CM) [5, 6, 7]. Therefore, SM generally presents a poor prognosis with a high local recurrence rate and low median survival [8].

Metabolic reprogramming in the tumor microenvironment is crucial for the survival and even proliferation of cancer cells under stressful situations [9]. Lipid droplet (LD) accumulation, lipogenesis, and increased glycolysis have firmly been established as cancer‐associated metabolic changes to face harsh environments. LD accumulation has been reported to play a key role in protection against reactive oxygen species toxicity (ROS), cell survival, and tumor growth under hypoxic conditions [10, 11]. In the latter, adipophilin (ADRP‐ a well‐known LD coat protein) is one of the molecules that are essential for LD formation [10, 11]. Regarding lipogenesis, increased lipid synthesis contributes to cancer cell survival as well as tumor growth. Indeed, fatty acid synthase (FASN) is overexpressed in several cancers, and its inhibitors have been reported to reduce melanoma cell proliferation and activate the intrinsic pathway of apoptosis [11]. Besides these alterations of lipid metabolism, increased glycolytic capacity plays a key role in tumor survival through the induction of glucose transporters (GLUT) and several glycolytic enzymes. GLUT1 is one of the most studied glucose transporters and is overexpressed in a significant proportion of human malignancies [12].

The morphological particularities of SM led us to hypothesize that the environmental conditions could be more adverse in SM than in OM and CM, influencing tumor metabolism. Proteins related to the metabolic reprogramming of cancer cells have been considered potential therapeutic targets; but in SM, the expression of such proteins has yet to be explored. There are few studies on the immunohistochemical expression of the metabolic phenotype in melanomas. Understanding these two phenomena has potential importance to the development of new therapeutic targets since melanomas are notorious for their intrinsic resistance to chemotherapy. For this reason, this study aimed to compare the immunohistochemical expression of adipophilin, FASN, GLUT‐1, and HIF‐1α across three aggressive types of melanomas.

## 2 Materials and Methods

### 2.1 Tissue Samples

After approval by the Research Ethics Committee of the Faculty of Medical Sciences of the State University of Campinas (#1424893), 28 SM, 16 OM, and 39 CM were examined. Regarding CM, 25 of these cases were paired with their respective lymph node metastases (primary and metastatic tumor). As controls, 17 melanocytic nevi of the mucosa and 15 cutaneous melanocytic nevi were used for comparison. The diagnosis of melanoma was previously established by hematoxylin and eosin (HE) staining and, when necessary, by immunohistochemistry using antibodies to S‐100, HMB45, and Melan A protein.

### 2.2 Pathological Analysis

The histopathological criteria used for microscopic description were: (1) presence or absence of melanin (melanotic or amelanotic); (2) level of invasion of tumor cells in OM and SM according to the classification of Prasad et al. [13] (level I: melanoma in situ; level II: invasion of lamina propria, and level III: deep invasion of skeletal muscle, bone or cartilage); (3) level of invasion of tumor cells in CM according to the classification of Clark et al. [14] (level I: melanoma in situ; level II: partial invasion of papillary dermis; level III: total invasion of papillary dermis; level IV: invasion of reticular dermis; level V: invasion of subcutaneous tissue); (4) level of tumor thickness in non‐metastatic and metastatic CM with their respective metastases according to Breslow [15]: < 1 mm or > 1 mm; (5) pattern of cell organization and distribution (alveolar, organoid, pagetoid, solid); (6) predominant cell type in the tumor; (7) presence of necrosis; (8) perineural invasion; and (9) vascular invasion.

### 2.3 Immunohistochemical Analysis

For the immunohistochemical reaction, 3 μm thick sections of paraffin‐embedded tissues were made and placed on slides previously treated with 3‐aminopropyl‐triethoxy‐silane (Sigma Chemical Company, USA). Initially, the sections were deparaffinized, hydrated in alcohol baths, and subjected to antigen retrieval. Endogenous peroxidase blockade was performed using 10% hydrogen peroxide. Subsequently, the sections were incubated with the primary antibodies (overnight) and the secondary antibodies (Advance DAKO, for 1 h at 37°C.). Sections were stained with 3,3′‐diaminobenzidine tetrahydrochloride or Permanent Red (for FASN) and contrasted with Mayer hematoxylin. The primary antibodies, specifications, and antigenic retrieval method employed are described in Table S1.

### 2.4 Evaluation and Quantification of Positive Cells

The immunohistochemical reactions were interpreted simultaneously by two researchers (AA and JN). The evaluation of the positivity of the reaction was performed at low magnification (5×) to identify the areas of higher labeling density, and the following semiquantitative scale was used for adipophilin, FASN, GLUT‐1, and HIF‐1α antibodies: (0) when staining was absent or there was positivity < 5% in the examined cells; (+) when positivity was ≥ 5% and < 50% in the examined cells; and (++) when positivity was ≥ 50% in the examined cells. The cell proliferation index by Ki‐67 was calculated by the ratio of the number of positive cells to the total number of tumor cells, calculated using the Aperio ImageScope (Aperio ScanScope; Aperio Technologies, Vista, Calif). Five higher magnification fields were randomly selected (×200) and approximately 3000 tumor cells per slide were counted. The labeling index for Ki‐67 was expressed as the percentage of positive tumor cells according to Angelis et al. [16].

### 2.5 Statistical Analysis

The *χ* ^2^ test was used to find a value of the scatter between nominal and qualitative variables. Fisher's exact *χ* ^2^ test was used to evaluate the associations between categorical data. The Kruskal‐Wallis and Mann–Whitney tests were used to compare the numerical variables between the negative (0) and positive (+ and ++) groups. Statistical analysis was performed using MedCalc Statistical Software version 14.8.1 (MedCalc Software, Ostend, Belgium; http://www.medcalc.org; 2014). The significance level considered was 5% (*p* < 0.05) for all tests.

## 3 Results

### 3.1 Histopathological Analysis

**FIGURE 1:**
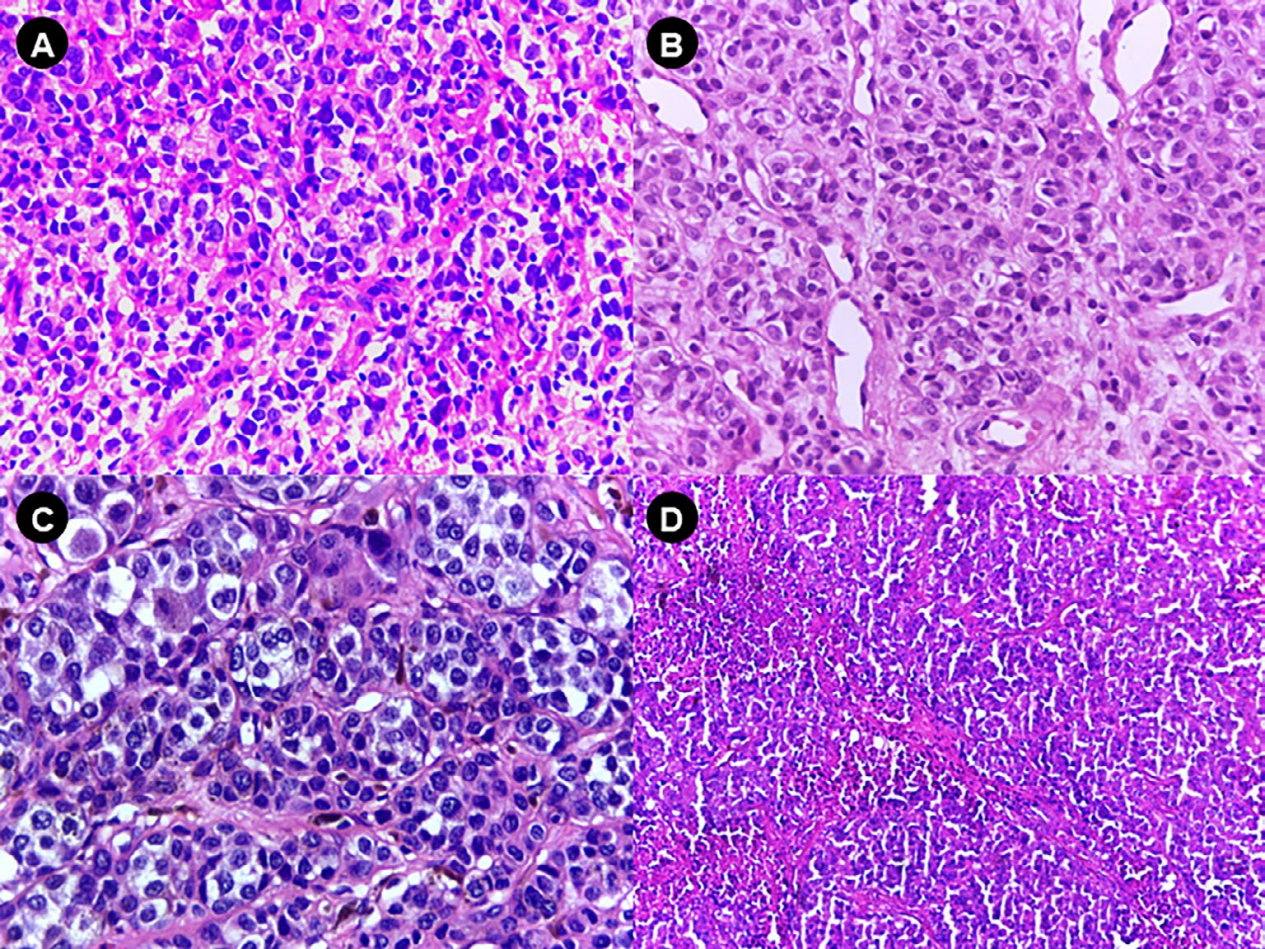
Histological features of melanomas under H&E staining. (A) Undifferentiated cells (small, round) in SM (original magnification, ×40). (B) Epithelioid cells in OM (original magnification, ×40). (C) Epithelioid cells in non‐metastatic melanomas (original magnification, ×40). (D) Undifferentiated cells (small, round) observed in metastatic melanomas (original magnification, ×10). OM, oral melanoma; SM, sinonasal melanoma.

**FIGURE 2:**
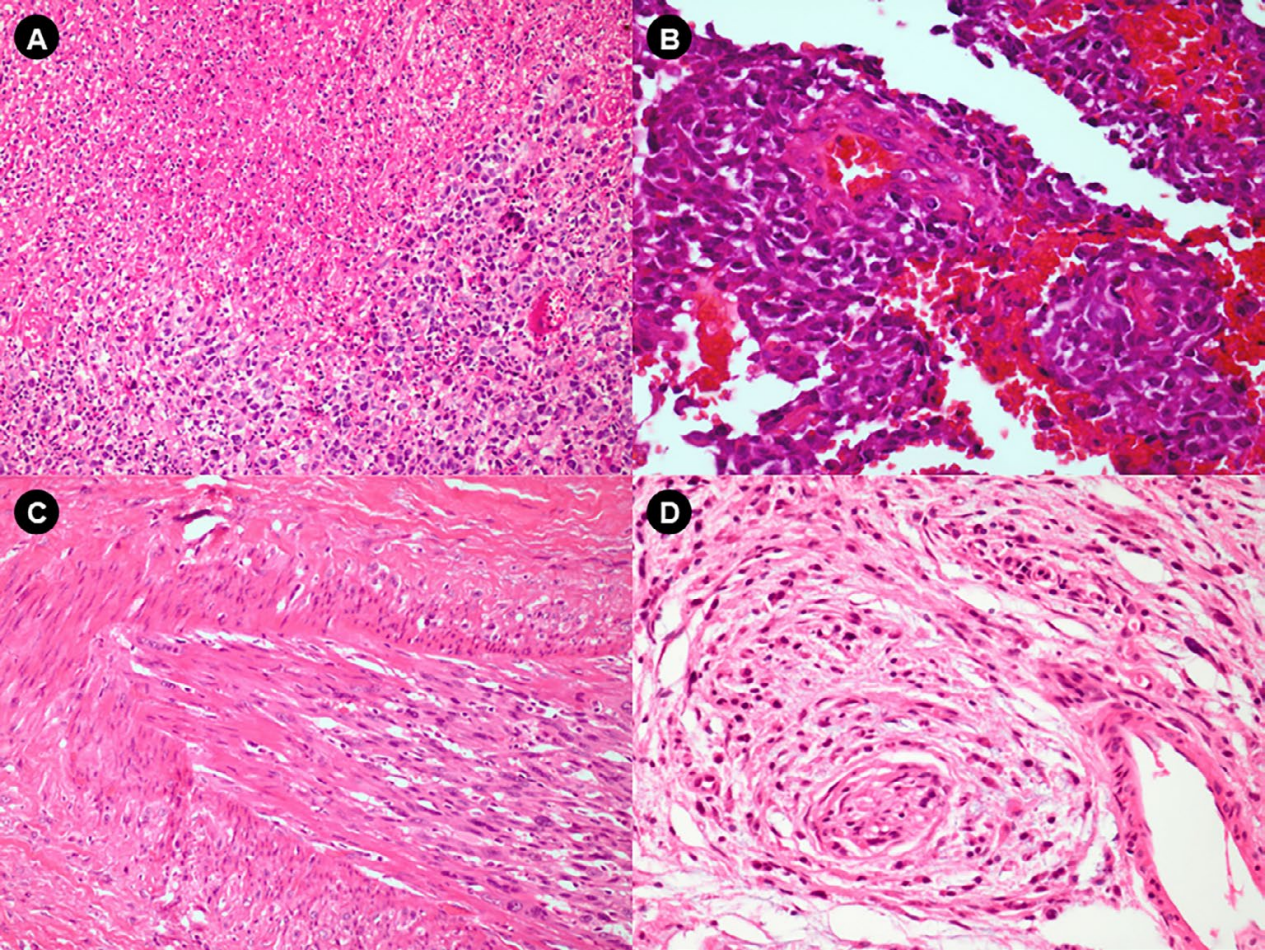
Necrosis and invasion patterns in SM and OM. (A) Necrotic areas associated with undifferentiated cells (small, round) in SM (original magnification, ×10). (B) Neoplastic cells forming a perivascular cuff in SM (original magnification, ×40). (C) Vascular invasion in SM (original magnification, ×40). (D) Neural invasion in OM (original magnification, ×40). OM, oral melanoma; SM, sinonasal melanoma.

SM was distinguished from OM by the high frequency of poorly differentiated morphological appearance, that is, neoplasm composed predominantly of small round cells (60% of SM cases vs. 12% of OM cases) (Figure 1A). The cellular composition of most OM was mainly epithelioid (Table S2, Figure 1B). Regarding CM, 39 cases were selected and subdivided into 14 non‐metastatic and 25 metastatic tumors with their respective lymph node metastases. In both groups, most tumors were > 1 mm thick (Table S3). Small and round cells were the main predominant cell types in the metastatic melanomas (in both primary lesions and metastases), while in the non‐metastatic group, it was the epithelioid cell type (Figure 1C,D). All 17 cases of nevi showed the nevus cells only in the connective tissue (intramucosal pattern) (Table S4, Figure 1E). As for melanogenesis, SM had a lower percentage of cases with marked melanogenesis (14.8% of cases with melanin in > 50% of cells) than OM (33.3%). Melanogenesis was more pronounced in the primary tumor (present in 68% of cases) than in the metastases (56%) (Table S5). Tumor necrosis was detected in almost all cases of SM (96%). In these cases, generally extensive and usually associated with the undifferentiated round cell pattern, with viable cells often surrounding the vessels (> 50% of the tumor in 57.1% of cases) (Figure 2, Table S5). In contrast, necrosis in OM was focal and infrequent (18% of cases) and lymph node metastases had a markedly higher frequency of necrosis (80%) compared to the primary tumor (28%), and in most cases (60%) it was extensive in the metastases (> 50% of the lesion) (Table S6).

### 3.2 SM Show Higher Expression of Adipophilin Compared With OM

**FIGURE 3:**
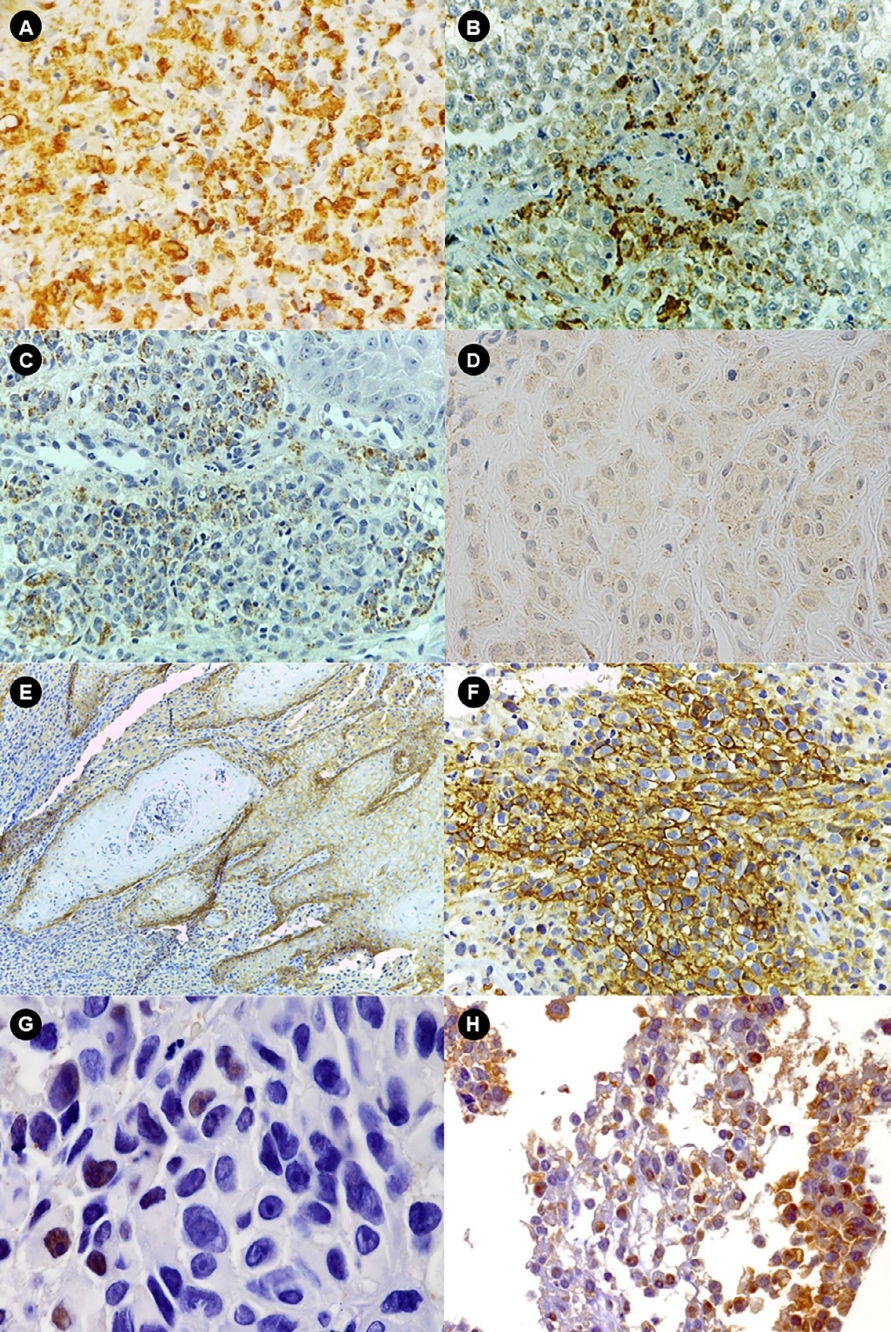
Immunohistochemical staining of melanomas for adipophilin, GLUT‐1, and HIF1‐α. (A) Adipophilin expression in SM (original magnification, ×40). (B) Adipophilin positivity around perinecrotic areas in SM (original magnification, ×40). (C) Adipophilin expression in OM (original magnification, ×40). (D) Adipophilin staining in oral nevi (original magnification, ×40). (E) GLUT‐1 expression in OM (original magnification, ×10). (F) GLUT‐1 expression in perinecrotic areas of SM (original magnification, ×40). (G) Focal HIF1‐α expression in OM (original magnification, ×40). (H) Diffuse HIF1‐α expression in SM (original magnification, ×20). OM, oral melanoma; SM, sinonasal melanoma.

Adipophilin was observed in neoplastic cells involving tiny cytoplasmic LD. SM showed the highest frequency of adipophilin expression (92.8%), and in most cases, it was diffused (> 50% of cells); in 67.8%, there was a greater accumulation of LD in the perinecrotic areas (Figure 3A,B). The frequency of adipophilin expression in SM was significantly higher than in OM (92.8% vs. 62.5% of cases). In OM, the perinecrotic pattern of distribution of adipophilin‐positive LD was not noted, except in 3 cases that showed marked accumulation in the neoplastic cells near the ulcerated superficial areas (Figure 3C). As for benign melanocytic lesions, adipophilin‐positive LD were rarely observed in oral nevi (1/17 cases) (Figure 3D). SM showed the highest frequency of GLUT‐1 expression (38.9% of cases), but the difference was not statistically significant compared to OM (20% of cases) (Figure 3E,F). In all cases, the pattern of GLUT‐1 expression was perinecrotic. In oral benign melanocytic lesions, no GLUT‐1 expression was detected. With regards to HIF‐1α, the nuclear expression of the protein was higher in SM—37.5% than in OM—20%, but the difference between them was not significant (Figure 3G) (*p* = 0.6). In SM, in which characteristically the peritheliomatous pattern (cell cuff surrounding a vessel) is observed, most of the HIF‐1α positive cells were located at the periphery of the tumor (Figure 3H) (Table 1). The nuclear expression of HIF‐1α was noted in only 1 case of nevus and a focal pattern.

**TABLE 1:** Distribution of FASN expression level in melanomas classified according to the site of origin.

Groups	0	1+	2+	Total	Statistical testing
	No. (%)	No. (%)	No. (%)	No. (%)	*χ* ^2^
Adipophilin					
OM	6 (37.5)	4 (25)	6 (37.5)	16 (100)	*p =* 0.018
SM	2 (7.1)	4 (14.3)	22 (78.6)	28 (100)	
GLUT‐1					
OM	8 (80)	2 (20)	0 (0)	10 (100)	*p =* 0.448
SM	11 (61.1)	5 (27.8)	2 (11.1)	18 (100)	
FASN					
OM	3 (18.8)	8 (50)	5 (31.3)	16 (100)	*p =* 0.872
SM	5 (19.2)	11 (42.3)	10 (38.5)	26 (100)	
HIF‐1α					
OM	8 (80)	2 (20)	0 (0)	10 (100)	*p =* 0.6
SM	18 (64.2)	9 (32.1)	1 (3.5)	28 (100)	

Abbreviations: OM, oral melanoma; SM, sinonasal melanoma.

### 3.3 Primary Tumor and Metastases Did Not Exhibit Significant Modification in Adipophilin/LD and HIF‐1α Expression

**FIGURE 4:**
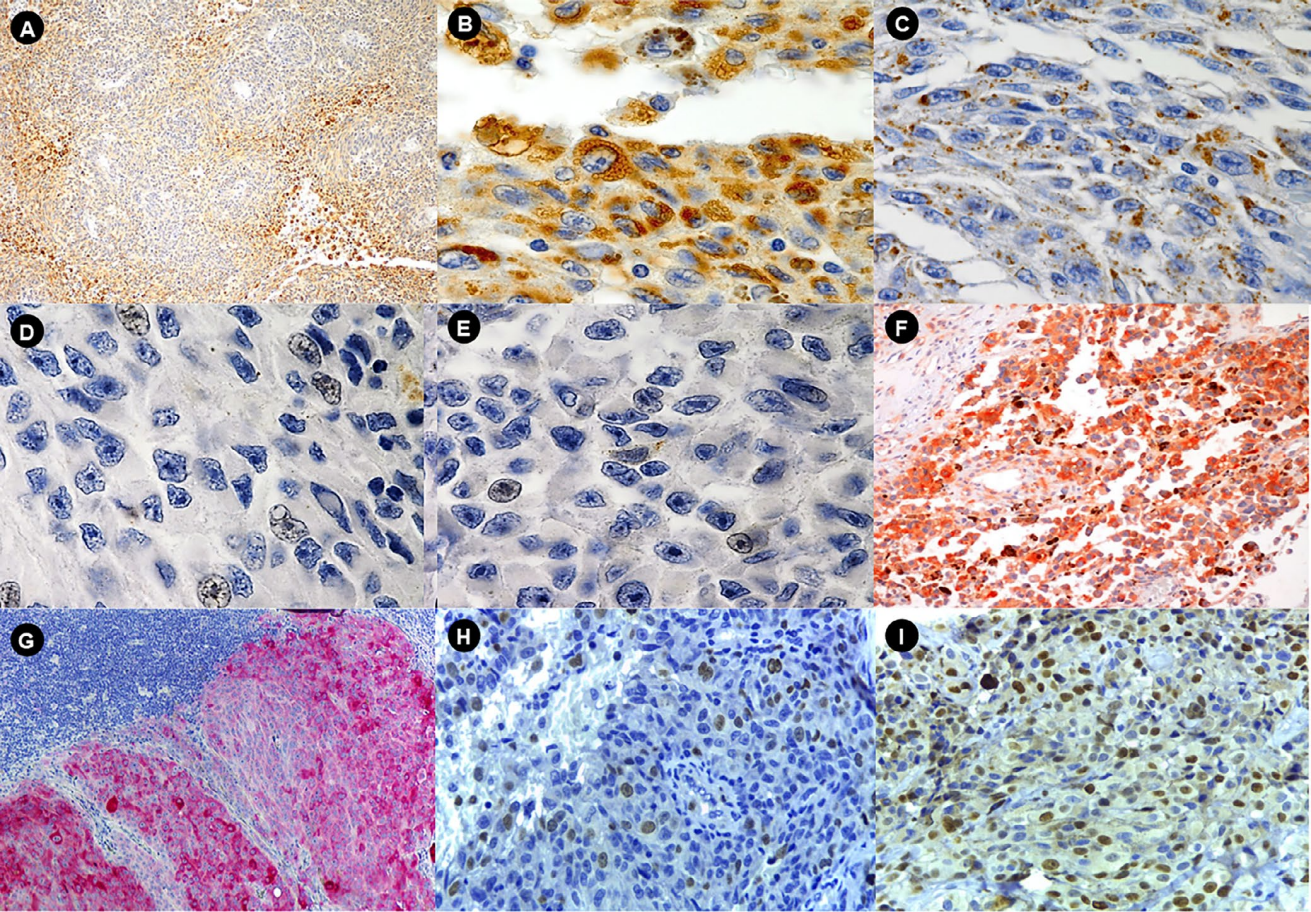
Immunohistochemical analysis of adipophilin, HIF1‐α, FASN, and Ki‐67 in primary and metastatic melanomas. (A, B) Adipophilin expression in lymph node metastases (original magnifications, ×10 and ×40). (C) Adipophilin positivity in primary cutaneous melanoma (original magnification, ×40). (D) HIF1‐α expression in primary cutaneous melanoma (original magnification, ×40). (E) Focal HIF1‐α expression in lymph node metastases (original magnification, ×40). (F) Diffuse FASN expression in SM (original magnification, ×20). (G) FASN expression in lymph node metastases (original magnification, ×40). (H) Ki‐67 expression in SM (original magnification, ×40). (I) Ki‐67 expression in OM (original magnification, ×40). OM, oral melanoma; SM, sinonasal melanoma.

The expression of adipophilin enabled the evaluation of the accumulation of intracytoplasmic LD, and these were found in both nevi and melanomas (non‐metastatic, metastatic, and lymph node metastases). However, although there was an increase in the amount of LD in melanomas, the difference was not significant (*p* = 0.19). Additionally, perinecrotic accentuation of LD accumulation was noted in melanomas, that is, the viable neoplastic cells surrounding the necrosis contained numerous LD. As expected, this perinecrotic accentuation of LD occurred particularly in lymph node metastases (40% of cases), since these had a higher frequency of necrosis. Comparing the primary tumor with its respective lymph node metastasis, the pattern of adipophilin expression remained the same in 64% of metastatic cases, increased by 20%, and decreased by 16% in these metastatic cases (Figure 4A–C). In melanomas, positivity for HIF‐1α was observed in less than 50% of metastatic and non‐metastatic cases, and almost all of them were in focal patterns (Figure 4D,E, Table 2).

**TABLE 2:** Distribution of Adipophilin, FASN, and HIF‐1α expression levels in non‐metastatic melanomas, metastatic melanomas, and cutaneous nevi.

Groups	Adipophilin			*p* *		FASN		*p* *		HIF‐1α		*p* *
	“0”	“+”	“++”		“0”	“+”	“++”		“0”	“+”	“++”	
	*n* (%)	*n* (%)	*n* (%)		*n* (%)	*n* (%)	*n* (%)		*n* (%)	*n* (%)	*n* (%)	
Cutaneous nevus	8 (53.3)	3 (−20)	4 (36.7)		10 (66.7)	4 (26.4)	1 (6.7)		14 (93.3)	1 (6.7)	0	
Non‐metastatic melanomas	3 (21.4)	5 (35.4)	6 (42.9)		3 (27.3)	2 (18.2)	6 (54.5)^a^		12 (85.7)	1 (7.1)	1 (7.1)^a^	
Metastatic melanomas				0.19				< 0.0001				0.03
Primary	5 (−20)	12 (−48)	8 (−32)		0	1 (−4)	24 (96)^b^		14 (58.3)	9 (37.5)	01 (4.2)^b^	
Lymph node metastases	6 (−24)	7 (−28)	12 (−48)		0	1 (−4)	24 (96)^c^		15 (62.5)	9 (37.5)	0 (00)^c^	

*Note*: 0: expression level absent, +: positivity ≤ 50% (focal positive), ++ > 50% (diffuse positive).

* *χ* ^2^ statistic test/*p*‐value FASN: ^a×b^ = 0.006, ^b×c^ = 1.00; HIF‐1α: ^a×b^ = 0.12, ^b×c^ = 0.59.

### 3.4 FASN Increases Its Expression in Mucosal Melanomas, Advanced CM, and Metastatic Tumors and Is Absent or Relatively Low in Benign Melanocytic Lesions

Most mucosal melanomas (> 80% of cases), regardless of the site of origin, showed FASN expression (Figure 4F, Table 1). In contrast, most benign melanocytic lesions did not show FASN expression; this was detected in 29.4% of oral nevi. FASN was only found in 4 cases (23%) of oral nevus, and its intensity was considered strong.

Metastatic and non‐metastatic melanomas showed a marked increase in FASN expression compared to cutaneous nevi. Furthermore, FASN expression was significantly increased in metastatic melanomas when compared to non‐metastatic melanomas (*p* = 0.006); but no significant difference was noted between primary tumor and lymph node metastasis (Figure 4G) (*p* = 1.00). As for the intensity of FASN, a strong intensity like that of control tissue (sebaceous gland or adipose tissue) was detected in a minority of metastatic (36% of primary melanomas and 28% of metastases) and non‐metastatic melanomas (14.2%). The metastatic lesion showed maintenance of expression extent relative to the primary tumor in 13 cases (52%), an increase in 3 (12%), and a decrease in 9 (36%) (Table 2).

### 3.5 Metastatic CM Have a Significantly Lower Proliferative Index Than Non‐Metastatic Melanomas

The expression of Ki‐67 was absent in all cells of the oral nevi; however, it was present in the basal layer where cell proliferation occurs. There was no significant difference between the proliferative index of SM (mean 15.02) and OM (mean 18.86) (Figure 4H,I). The mean Ki‐67 proliferation index was 19.97 in non‐metastatic melanomas, decreasing significantly in metastatic melanomas—14.40 (*p* = 0.0001). However, the difference between the proliferative index of the primary tumor and lymph node metastasis was not significant (*p* = 0.2) (Table 3).

**TABLE 3:** KI‐67 proliferation index in mucosal melanomas, non‐metastatic cutaneous melanomas, and metastatic melanomas.

Groups	*N*	Average	Standard deviation	Min.	Max.	Total (%)	*p*
Oral melanoma	14	18.86	11.5	5	43	28	0.424*
Sinonasal melanoma	24	15.02	7.45	0	30	48	
Non‐metastatic cutaneous melanoma^a^	9	19.97	11.0	1.7	44.3	15.5	
Metastatic melanomas							
Primary^b^	24	14.40	8.4	3.6	32.7	41.3	0.02**
Lymph node metastases^c^	25	10.66	10.9	1	36.8	43.2	
Total	58	14	10.2	1	44.3	100	

* Mann–Whitney test.

** Kruskal‐Wallis test/*p*‐value: ^a×b^ = < 0.0001; ^b×c^: 0.2.

## 4 Discussion

In recent decades, the reprogramming of cellular metabolism by oncogenes has been a vital element in the advancement of melanoma. Metabolic reprogramming is crucial for melanoma cells to transition between different phenotypic states needed for surviving in nutrient‐deprived microenvironments or hypoxia [17, 18]. In the current study, we showed that SM presented distinguishable features from OM and CM, such as pseudopapillary growth pattern, the predominance of undifferentiated morphology, low melanogenesis, high frequency of necrosis, and marked accumulation of adipophilin‐positive LD. OM and CM exhibited similarities in tumor morphology, expression of adipophilin, FASN, and GLUT1, and Ki‐67 proliferation index.

Our findings reinforce the hypothesis proposed by Prasad et al. (2004) [13] that the native microenvironment of the ciliated respiratory mucosa has possible implications in the more aggressive features of SM. Structural characteristics of respiratory mucosa (a looser and more richly vascularized tissue than the one covered by squamous epithelium) have been thought to be an explanation for the usual polypoid appearance of SM that would be associated with a higher frequency of ulceration and necrosis. However, the predominance of undifferentiated cellular morphology and marked LD accumulation in SM suggests that more complex factors may be involved in the pathogenesis of this aggressive lesion. In terms of diagnosis, awareness of the undifferentiated morphology of SM is important given that the region is frequently affected by other malignancies composed of small blue cells.

Histologically, the presence of goblet cells in the mucosal epithelium of the upper airway is closely associated with the production of cytokines such as IL‐13, IL‐8, and IL‐9, which play a role in inflammatory pathways and consequently tumor progression. In addition, studies have demonstrated the involvement of these cells in tumor development in other regions of the body, such as the intestine [19, 20]. Their role in tumor initiation and progression in the sinonasal region requires further investigation. However, the importance of the inflammatory pathway, particularly as a potential aid to tumor suppression, cannot be dismissed.

LD are dynamic organelles originating from endoplasmic reticulum membranes; they are composed of a central core of neutral lipids, surrounded by a monolayer of phospholipids that contains several proteins, among them adipophilin. LD is ubiquitously present in eukaryotic cells and has been associated with energy regulation, fatty acid storage, membrane biosynthesis, and control of lipid signaling molecules. The LD levels can increase following cellular stress (including hypoxia, induction of apoptosis by different insults, contact inhibition, inflammation) or lipid overload [21, 22, 23, 24]. In malignancies, LD accumulation has been reported as a common phenomenon that may be associated with tumor differentiation, proliferation, aggressiveness, and, more recently, an emerging therapeutic target [25, 26, 27, 28, 29, 30]. In the current series, SMs presented a marked difference regarding LD levels when compared to OM and CM. In most SM cases, LD was observed in a diffuse pattern in tumor cells (> 50%). Furthermore, accentuation of LD accumulation in perinecrotic areas was also seen in 68% of lesions. In contrast, OM and CM showed less frequent LD diffuse patterns, and their accumulation in perinecrotic areas was rarer. LD accumulation in perinecrotic areas is an expected finding since such a phenomenon has been considered a surrogate marker of severe, perinecrotic hypoxia [31]. In vitro studies using cell lines to study metabolic pathways, combined with in vivo assays to study the tumor microenvironment interactions, are essential for understanding metabolic reprogramming. In addition, incorporating metabolic profiling and treatment response correlations into these studies will provide valuable insights and pave the way for advances in personalized medicine.

In tumor cell lines, an inverse correlation between O_2_ concentration and LD levels has been observed, and lipid storage has been suggested to be essential for cell recovery after the restoration of normoxic levels [10]. However, the significantly higher LD accumulation in non‐perinecrotic areas in SNM can be triggered by diverse factors other than hypoxia. LD accumulation in neoplastic cells has also been linked to tumor differentiation and proliferation, but there is no evidence for these associations in our cases (Ki‐67 proliferation index in SM was not significantly different from the OM and CM). In SM, LD levels did not differ between tumors composed of undifferentiated small cells and the ones containing other cell types.

FASN is widely known that this crucial enzyme for fatty acid synthesis [32, 33, 34] has been correlated to malignant phenotype in different tumors [16, 28, 32, 34, 35, 36, 37, 38]. Here, in the CM and their metastases, we showed that advanced CMs express FASN more often than benign lesions, and there is a significant increase of FASN expression in the tumors that metastasize. Innocenzi et al. [39] analyzing a series of CM and their metastases reported that patients with a higher FASN expression had a higher risk of developing recurrences and metastases than those with lower expression. Saab et al. showed that all metastatic melanomas involving sentinel lymph nodes were FASN positive. In contrast, benign intracapsular nevi in sentinel lymph nodes of patients with CM did not show FASN expression [40].

In the field of targeted therapy, experimental studies have described that FASN inhibitors reduce tumor progression in other tumors [41, 42, 43, 44, 45, 46] and could reduce metastasis in models of melanoma cells [44, 45]. Curiously, FASN inhibition can activate the intrinsic apoptosis pathway by mitochondrial involvement in melanoma cells [11]. Previous results from our group showed that mitochondrial markers could play an important role during multiple stages of tumorigenesis and in the development of metastases of CM and mucosal melanomas [46]. However, further studies with larger cohorts are needed to validate FASN as a reliable prognostic marker. While the results are promising, it is important to recognize that the tumor biology of the samples may have been influenced by several factors, including patient demographics, treatment history, and environmental or geographic conditions. In particular, the high genetic diversity observed in Latin American populations may also contribute to this variability.

## 5 Conclusion

Here, we showed that the marked expression of FASN in metastatic CMs suggests that FASN may be involved in disease progression. SM presents a pronounced expression of adipophilin compared to OM. The tendency toward increased expression of GLUT‐1 and HIF‐1α, together with the extensive necrosis in SM, suggests that its microenvironment may be more hypoxic, which could at least partially explain the higher accumulation of adipophilin‐positive LD observed in this tumor.

## Author Contributions

J.S.N., J.F.S., A.A., and F.V.M. conceived and designed the study. J.S.N., J.F.S., and E.S.A.E. performed the immunohistochemical and pathological analyses. M.B.C. and R.R.D.R. contributed with sample acquisition and clinical data. L.S.A. and R.M.M.R. participated in the histological evaluation. C.A.L.P. and P.M.P. provided support in interpreting pathological data. A.L.N.F., F.P.F., and L.P.K. contributed with scientific review and manuscript editing. R.C. contributed significantly in the early phases of the project and in the collection of clinical samples. A.A. and J.S.N. performed the immunohistochemical scoring. J.S.N. and J.F.S. performed the statistical analysis and drafted the first version of the manuscript. All authors reviewed and approved the final version of the manuscript.

## Ethics Statement

The present study was approved by the Research Ethics Committee of the Faculty of Medical Sciences of the State University of Campinas (1424893) and conducted in accordance with the Declaration of Helsinki. No identifiable information is contained in the data. There is no material from other sources in this submission.

## Conflicts of Interest

The authors declare no conflicts of interest.

## Supporting information


**Data S1:** Supporting Information.

## Data Availability

The data that support the findings of this study are available from the corresponding author upon reasonable request.

## References

[1] D. Holmes , “The Cancer That Rises With the Sun,” Nature 515 (2014): S110–S111. 25407705 10.1038/515S110a

[2] V. J. Lund , “Sinonasal Malignant Melanoma,” Advances in Otorhinolaryngology 84 (2020): 185–196. 10.1159/000457937 32731237

[3] M. Boulaadas , S. Benazzou , F. Mourtada , et al., “Primary Oral Malignant Melanoma,” Journal of Craniofacial Surgery 18 (2007): 1059–1061. 17912082 10.1097/scs.0b013e3180f6120e

[4] “Key Statistics for Melanoma Skin Cancer,” accessed August 20, 2025, https://www.cancer.org/cancer/types/melanoma‐skin‐cancer/about/key‐statistics.html.

[5] M. L. Prasad , K. J. Busam , S. G. Patel , et al., “Clinicopathologic Differences in Malignant Melanoma Arising in Oral Squamous and Sinonasal Respiratory Mucosa of the Upper Aerodigestive Tract,” Archives of Pathology & Laboratory Medicine 127 (2003): 997–1002. 12873174 10.5858/2003-127-997-CDIMMA

[6] J. F. Thompson , R. A. Scolyer , and R. F. Kefford , “Cutaneous melanoma,” Lancet 365 (2005): 687–701. 15721476 10.1016/S0140-6736(05)17951-3

[7] G. Cazzato , “Histopathological Diagnosis of Malignant Melanoma at the Dawn of 2023: Knowledge Gained and New Challenges,” Dermatopathology 10 (2023): 91–92. 36810571 10.3390/dermatopathology10010013 PMC9944108

[8] F. S. C. Pontes , L. L. de Souza , M. C. de Abreu , et al., “Sinonasal Melanoma: A Systematic Review of the Prognostic Factors,” International Journal of Oral and Maxillofacial Surgery 49 (2020): 549–557. 31767512 10.1016/j.ijom.2019.11.001

[9] S. Nong , X. Han , Y. Xiang , et al., “Metabolic Reprogramming in Cancer: Mechanisms and Therapeutics,” MedComm 4 (2023): e218. 36994237 10.1002/mco2.218 PMC10041388

[10] K. Bensaad and A. L. Harris , “Hypoxia and Metabolism in Cancer,” in Tumor Microenvironment and Cellular Stress: Signaling, Metabolism, Imaging, and Therapeutic Targets, ed. C. Koumenis , E. Hammond , and A. J. Giaccia (Springer, 2014), 1–39. 10.1007/978-1-4614-5915-6 24765667

[11] K. G. Zecchin , F. A. Rossato , H. F. Raposo , et al., “Inhibition of Fatty Acid Synthase in Melanoma Cells Activates the Intrinsic Pathway of Apoptosis,” Laboratory Investigation 91 (2011): 232–240. 20805790 10.1038/labinvest.2010.157

[12] J. Wang , C. Ye , C. Chen , et al., “Glucose Transporter GLUT1 Expression and Clinical Outcome in Solid Tumors: A Systematic Review and Meta‐Analysis,” Oncotarget 8 (2017): 16875–16886. 28187435 10.18632/oncotarget.15171 PMC5370007

[13] M. L. Prasad , S. G. Patel , A. G. Huvos , et al., “Primary Mucosal Melanoma of the Head and Neck,” Cancer 100 (2004): 1657–1664. 15073854 10.1002/cncr.20201

[14] W. H. Clark, Jr. , “A Classification of Malignant Melanoma in Man Correlated With Histogenesis and Biologic Behavior,” Advances in the Biology of the Skin 8 (1967): 621–647.

[15] A. Breslow , “Thickness, Cross‐Sectional Areas and Depth of Invasion in the Prognosis of Cutaneous Melanoma,” Annals of Surgery 172 (1970): 902–908. 5477666 10.1097/00000658-197011000-00017 PMC1397358

[16] C. M. de Angelis , R. A. de Lima‐Souza , J. F. Scarini , et al., “Immunohistochemical Expression of Fatty Acid Synthase (FASN) is Correlated to Tumor Aggressiveness and Cellular Differentiation in Salivary Gland Carcinomas,” Head and Neck Pathology 15 (2021): 1119–1126. 33843033 10.1007/s12105-021-01319-3 PMC8633252

[17] P. Falletta , C. R. Goding , and Y. Vivas‐García , “Connecting Metabolic Rewiring With Phenotype Switching in Melanoma,” Frontiers in Cell and Development Biology 10 (2022): 10. 10.3389/fcell.2022.930250 PMC9334657 35912100

[18] M. Kuras , “Exploring the Complex and Multifaceted Interplay Between Melanoma Cells and the Tumor Microenvironment,” International Journal of Molecular Sciences 24 (2023): 14403. 37762707 10.3390/ijms241814403 PMC10531837

[19] S. Siddiqui , K. Johansson , A. Joo , et al., “Epithelial miR‐141 Regulates IL‐13‐Induced Airway Mucus Production,” JCI Insight 6 (2021): e139019. 33682796 10.1172/jci.insight.139019 PMC8021117

[20] C. Yuan , A. Rayasam , A. Moe , et al., “Interleukin‐9 Production by Type 2 Innate Lymphoid Cells Induces Paneth Cell Metaplasia and Small Intestinal Remodeling,” Nature Communications 14 (2023): 7963. 10.1038/s41467-023-43248-5 PMC10693577 38042840

[21] K. Bensaad , E. Favaro , C. A. Lewis , et al., “Fatty Acid Uptake and Lipid Storage Induced by HIF‐1α Contribute to Cell Growth and Survival After Hypoxia‐Reoxygenation,” Cell Reports 9 (2014): 349–365. 25263561 10.1016/j.celrep.2014.08.056

[22] R. V. Farese and T. C. Walther , “Lipid Droplets Finally Get a Little R‐E‐S‐P‐E‐C‐T,” Cell 139 (2009): 855–860. 19945371 10.1016/j.cell.2009.11.005 PMC3097139

[23] B. K. Straub , B. Gyoengyoesi , M. Koenig , et al., “Adipophilin/Perilipin‐2 as a Lipid Droplet‐Specific Marker for Metabolically Active Cells and Diseases Associated With Metabolic Dysregulation,” Histopathology 62 (2013): 617–631. 23347084 10.1111/his.12038

[24] A. Gubern , M. Barceló‐Torns , J. Casas , et al., “Lipid Droplet Biogenesis Induced by Stress Involves Triacylglycerol Synthesis That Depends on Group VIA Phospholipase A2,” Journal of Biological Chemistry 284 (2009): 5697–5708. 19117952 10.1074/jbc.M806173200

[25] B. K. Straub , E. Herpel , S. Singer , et al., “Lipid Droplet‐Associated PAT‐Proteins Show Frequent and Differential Expression in Neoplastic Steatogenesis,” Modern Pathology 23 (2010): 480–492. 20081801 10.1038/modpathol.2009.191

[26] H. T. dos Santos , R. N. Silva , A. R. Piña , et al., “Lipid Droplets Are Involved in the Process of High‐Grade Transformation of Adenoid Cystic Carcinoma,” Histopathology 69 (2016): 160–162. 26648334 10.1111/his.12916

[27] F. V. Mariano , H. T. dos Santos , W. D. Azañero , et al., “Mammary Analogue Secretory Carcinoma of Salivary Glands Is a Lipid‐Rich Tumour, and Adipophilin Can Be Valuable in Its Identification,” Histopathology 63 (2013): 558–567. 23931576 10.1111/his.12192

[28] J. F. Scarini , L. F. Rosa , R. A. d. L. Souza , et al., “Gene and Immunohistochemical Expression of HIF‐1α, GLUT‐1, FASN, and Adipophilin in Carcinoma ex Pleomorphic Adenoma Development,” Oral Diseases 26 (2020): 1190–1199. 32180291 10.1111/odi.13332

[29] D. Delmas , A. K. Cotte , J.‐L. Connat , et al., “Emergence of Lipid Droplets in the Mechanisms of Carcinogenesis and Therapeutic Responses,” Cancers (Basel) 15 (2023): 4100. 37627128 10.3390/cancers15164100 PMC10452604

[30] Y. Jin , Y. Tan , J. Wu , and Z. Ren , “Lipid droplets: a cellular organelle vital in cancer cells,” Cell Death Discovery 9 (2023): 254. 37474495 10.1038/s41420-023-01493-z PMC10359296

[31] S. Zoula , P. F. J. W. Rijken , J. P. W. Peters , et al., “Pimonidazole Binding in C6 Rat Brain Glioma: Relation With Lipid Droplet Detection,” British Journal of Cancer 88 (2003): 1439–1444. 12778075 10.1038/sj.bjc.6600837 PMC2741029

[32] F. P. Kuhajda , “Fatty‐Acid Synthase and Human Cancer: New Perspectives on Its Role in Tumor Biology,” Nutrition 16 (2000): 202–208. 10705076 10.1016/s0899-9007(99)00266-x

[33] R. Flavin , S. Peluso , P. L. Nguyen , and M. Loda , “Fatty Acid Synthase as a Potential Therapeutic Target in Cancer,” Future Oncology 6 (2010): 551–562. 20373869 10.2217/fon.10.11 PMC3197858

[34] J. A. Menendez and R. Lupu , “Fatty Acid Synthase and the Lipogenic Phenotype in Cancer Pathogenesis,” Nature Reviews. Cancer 7 (2007): 763–777. 17882277 10.1038/nrc2222

[35] R. A. de Lima‐Souza , N. d. M. Rodrigues , J. F. Scarini , et al., “Metabolic Alterations in Carcinoma ex Pleomorphic Adenoma Development of Lacrimal Glands,” International Ophthalmology 42 (2022): 1101–1109. 34757565 10.1007/s10792-021-02096-2

[36] R. Flavin , G. Zadra , and M. Loda , “Metabolic Alterations and Targeted Therapies in Prostate Cancer,” Journal of Pathology 223 (2011): 284–295. 10.1002/path.2809 PMC3197856 21125681

[37] L. Chang , S. Fang , Y. Chen , et al., “Inhibition of FASN Suppresses the Malignant Biological Behavior of Non‐Small Cell Lung Cancer Cells via Deregulating Glucose Metabolism and AKT/ERK Pathway,” Lipids in Health and Disease 18 (2019): 118. 31122252 10.1186/s12944-019-1058-8 PMC6533754

[38] T. Lu , L. Sun , Z. Wang , Y. Zhang , Z. He , and C. Xu , “Fatty Acid Synthase Enhances Colorectal Cancer Cell Proliferation and Metastasis via Regulating AMPK/mTOR Pathway,” Oncotargets and Therapy 12 (2019): 3339–3347. 31118685 10.2147/OTT.S199369 PMC6504633

[39] D. Innocenzi , P. L. Alò , A. Balzani , et al., “Fatty Acid Synthase Expression in Melanoma,” Journal of Cutaneous Pathology 30 (2003): 23–28. 12534800 10.1034/j.1600-0560.2003.300104.x

[40] J. Saab , M. L. Santos‐Zabala , M. Loda , E. C. Stack , and T. J. Hollmann , “Fatty Acid Synthase and Acetyl‐CoA Carboxylase Are Expressed in Nodal Metastatic Melanoma but Not in Benign Intracapsular Nodal Nevi,” American Journal of Dermatopathology 40 (2018): 259–264. 28654463 10.1097/DAD.0000000000000939 PMC6844149

[41] Y. Yoshii , T. Furukawa , N. Oyama , et al., “Fatty Acid Synthase Is a Key Target in Multiple Essential Tumor Functions of Prostate Cancer: Uptake of Radiolabeled Acetate as a Predictor of the Targeted Therapy Outcome,” PLoS One 8 (2013): e64570. 23741342 10.1371/journal.pone.0064570 PMC3669310

[42] H.‐W. Chen , Y.‐F. Chang , H.‐Y. Chuang , W. T. Tai , and J. J. Hwang , “Targeted Therapy With Fatty Acid Synthase Inhibitors in a Human Prostate Carcinoma LNCaP/Tk‐Luc‐Bearing Animal Model,” Prostate Cancer and Prostatic Diseases 15 (2012): 260–264. 22565411 10.1038/pcan.2012.15

[43] I. G. de Aquino , D. C. Bastos , F. J. M. Cuadra‐Zelaya , et al., “Anticancer Properties of the Fatty Acid Synthase Inhibitor TVB‐3166 on Oral Squamous Cell Carcinoma Cell Lines,” Archives of Oral Biology 113 (2020): 104707. 32197133 10.1016/j.archoralbio.2020.104707

[44] D. C. Bastos , J. Paupert , C. Maillard , et al., “Effects of Fatty Acid Synthase Inhibitors on Lymphatic Vessels: An in Vitro and in Vivo Study in a Melanoma Model,” Laboratory Investigation 97 (2017): 194–206. 27918556 10.1038/labinvest.2016.125

[45] F. Seguin , M. A. Carvalho , D. C. Bastos , et al., “The Fatty Acid Synthase Inhibitor Orlistat Reduces Experimental Metastases and Angiogenesis in B16‐F10 Melanomas,” British Journal of Cancer 107 (2012): 977–987. 22892389 10.1038/bjc.2012.355 PMC3464771

[46] C. D. Soares , T. M. Morais , F. V. Mariano , et al., “Expression of Mitochondrial Dynamics Markers During Melanoma Progression: Comparative Study of Head and Neck Cutaneous and Mucosal Melanomas,” Journal of Oral Pathology & Medicine 48 (2019): 373–381. 30916813 10.1111/jop.12855

