# Supplementary material for: Metabolic Heterogeneity Confers Differences in the Tumor Microenvironment of Aggressive Types of Melanomas
Source: J Oral Pathol Med. 2025 Aug 27;54(10):953–62. doi: 10.1111/jop.70042 (PMC12602135; doi:10.1111/jop.70042)
Supplement: Supplementary file 1 — Data S1: Supporting Information. [file JOP-54-953-s001.docx]

**Metabolic heterogeneity confers differences in the tumor microenvironment of aggressive types of melanomas**

**Supplemental Data**

| **Supplemental Data - Table 1** Primary antibodies, clones, dilution and manufacturers used in immunohistochemical analysis   \| **Primary antibody** \| **Clone** \| **Dilution** \| **Manufacturer** \| \| --- \| --- \| --- \| --- \| \| Adipophilin \| AP25 \| No dilution \| FITZGERALD \| \| FASN \| 2194 \| 1:100 \| SIGMA \| \| HIF-1α \| EP1215 \| 1:800 \| ABCAM \| \| GLUT-1 \| AB652 \| 1:200 \| ABCAM \| \| NANOG \| NNG-811 \| 1:100 \| SIGMA \| \| Ki-67 \| MIB-1 \| 1:100 \| DAKO \|   **Supplemental Data - Table 2** Predominant cell morphology in mucosal melanomas | | |
| --- | --- | --- | --- | --- | --- | --- | --- | --- | --- | --- | --- | --- | --- | --- | --- | --- | --- | --- | --- | --- | --- | --- | --- | --- | --- | --- | --- | --- | --- | --- |
|  |  |  |
| **Morphology** | **Mucosal melanoma** | |
| **Cell type** | **Oral melanoma** | **Sinonasal melanoma** |
| EC | 9/16 (56%) | 8/28 (29%) |
| SPC | 4/16 (25%) | 3/28 (11 %) |
| SC | 2/16 (12%) | 17/28 (60%) |
| PC | 1/16 (7%) | 0/28 (0%) |
| EC= Epithelioid cells; SPC= Spindle cells; SC= Small cells; PC= Plasmacytoid cells | | |

| \| **Supplemental Data - Table 3** Tumor thickness and invasion level of metastatic melanomas (MM) and non-metastatic melanomas (NMM) \| \| \| \| --- \| --- \| --- \| \|  \|  \|  \| \| **Variable** \| **MNM**  **n(%)** \| **MM**  **n(%)** \| \| **Breslow thickness (mm)** \|  \|  \| \| ≤ 1mm \| 3 (21) \| 1 (4) \| \| > 1mm \| 11(78) \| 21 (84) \| \| No information \| - \| 3 (12) \| \| **Clark's level of invasion** \|  \|  \| \| III \| 9 (64.2) \| 3 (12) \| \| IV \| 5 (35.8) \| 18 (72) \| \| V \| - \| 4 (16) \|  \| **Supplemental Data - Table 4** Predominant cell type in non-metastatic melanomas (NMM) and metastatic melanomas (MM) \| \| \| \| \| --- \| --- \| --- \| --- \| \|  \|  \|  \|  \| \| **Groups** \| **Predominant cell type** \| \| \| \| **SR***  **n(%)** \| **EP****  **n(%)** \| **SC*****  **n(%)** \| \| **MNM** \| 0 (0) \| 13 (92) \| 01 (8) \| \| **MM** \|  \|  \|  \| \| Primary \| 13 (52) \| 07 (28) \| 05 (20) \| \| Lymph node metastases \| 12 (48) \| 12 (48) \| 1 (4) \| \| *Small and round/ **Epithelioid/ ***Spindle cells \| \| \| \|   **Supplemental Data - Table 5** Melanogenesis and necrosis in mucosal melanomas | | | | |
| --- | --- | --- | --- | --- | --- | --- | --- | --- | --- | --- | --- | --- | --- | --- | --- | --- | --- | --- | --- | --- | --- | --- | --- | --- | --- | --- | --- | --- | --- | --- | --- | --- | --- | --- | --- | --- | --- | --- | --- | --- | --- | --- | --- | --- | --- | --- | --- | --- | --- | --- | --- | --- | --- | --- | --- | --- | --- | --- | --- | --- | --- | --- | --- | --- | --- | --- | --- | --- | --- | --- | --- | --- |
|  |  |  |  |  |
| **Oral melanoma** | | | **Sinonasal melanoma** | |
|  | **MG**** | **NC**** | **MG**** | **NC**** |
| **0** | 5/16 (31.2%) | 13/16 (81.2%) | 13/28 (46.4%) | 1/28 (3.5%) |
| **+** | 6/16 (37.6%) | 3/16 (18.7%) | 11/28 (39.2%) | 11/28 (39.2%) |
| **++** | 5/16 (31.2%) | 0/16 (0%) | 4/28 (14.2%) | 16/28 (57.1%) |
| **TOTAL** | 16/16 (100%) | 16/16 (100%) | 28/28 (100%) | 28/28 (100%) |
| 0: absent or <5% staining / +: positivity greater than ≥5% and <50% (focal positive) / ++: ≥ 50% (diffuse positive) | | | | |
| MG: Melanogenesis/ NC: Necrosis | | | | |

| **Supplemental Data - Table 6** Extent of necrosis (NC) and intensity of melanogenesis (MG) in metastatic primary melanomas (MPM) and lymph node metastases (LMM) | | | | |
| --- | --- | --- | --- | --- |
|  |  |  |  |  |
| **NC/MG*** | **NC/MPM** | **NC/LNM** | **MG/MPM** | **MG/MPM** |
|  | **n (%)** | **n (%)** | **n (%)** | **n (%)** |
| **0*** | 18 (72) | 5 (20) | 8 (32) | 11 (44) |
| **+**** | 3 (12) | 5 (20) | 6 (24) | 6 (24) |
| **++***** | 4 (16) | 15 (60) | 11 (44) | 8 (32) |
| * Absent/ **≤ 50% of tumor cells/ *** > 50% of tumor cells | | | | |
